# Cephalosporin-resistant *Escherichia coli* among Summer Camp Attendees with Salmonellosis

**DOI:** 10.3201/eid0910.030179

**Published:** 2003-10

**Authors:** Guillem Prats, Beatriz Mirelis, Elisenda Miró, Ferran Navarro, Teresa Llovet, James R. Johnson, Neus Camps, Ángela Domínguez, Lluis Salleras

**Affiliations:** * Universitat Autònoma, Barcelona, Spain; † University of Minnesota, Minneapolis, Minnesota, USA; ‡ Dept. de Sanitat i Seguretat Social, Barcelona, Spain

## Abstract

Investigation of an acute gastroenteritis outbreak involving >100 persons at a summer camp in Girona, Spain, in June 2002 led to the detection of *Salmonella* and extended-spectrum cephalosporin-resistant *Escherichia coli* (ESCREC). Stool cultures were performed for 22 symptomatic campers, three asymptomatic food handlers, and 10 healthy household members. Of the 22 campers, 19 had *Salmonella enterica*, 9 had an ESCREC strain carrying an extended-spectrum β-lactamase, and 2 had a second ESCREC strain carrying a plasmidic cephamycinase. Related ESCREC were detected in two (salmonella-negative) asymptomatic food handlers and in none of the healthy household members. Fecal ESCREC and its β-lactamases and plasmids were extensively characterized. Three of the five ESCREC clones were recovered from multiple hosts. The apparent dissemination of ESCREC suggests a food or water vehicle. The observed distribution of resistance plasmids and β-lactamase genes in several clones indicates a high degree of horizontal transfer. Heightened vigilance and increased efforts must be made to discover the reservoirs and vehicles for community dissemination of ESCREC.

Strains of *Escherichia coli* that produce enzymes capable of degrading extended- spectrum cephalosporins (ESCs), i.e., extended-spectrum β-lactamases (ESBLs), or these drugs plus cephamycins, i.e., plasmidic or hyperproduction of chromosomal cephamycinases have recently emerged as important nosocomial pathogens (*1*,*2*). Some of these strains cannot be reliably detected by clinical microbiology laboratories by using conventional susceptibility tests (*3*), and even when recognized, treating infections caused by these strains can be challenging because therapeutic options are limited. Infections attributable to such strains are associated with prolonged hospital stays, increased healthcare costs, and an increased number of deaths if appropriate therapy is delayed (*4*,*5*).

To date, almost all reports of infection or colonization with ESBL- and plasmidic cephamycinase-producing *E. coli* (i.e., extended-spectrum cephalosporin-resistant *E. coli* [ESCREC]) have involved hospitalized patients or nursing home residents (*3*,*6*). The few reported patients with community-acquired infection have been elderly and debilitated and have had hospital contact, important coexisting conditions, or both (*3*,*6*).

*E. coli*, including resistant strains, may be transmitted within the community through the food supply. Indeed, other gram-negative enteric pathogens, notably *Salmonella enterica*, are a frequent cause of foodborne disease and, increasingly, are associated with antibiotic resistance, including antibiotic resistance to ESCs (*7*–*11*). Available data regarding other resistant *E. coli* suggest that ESCREC could also be disseminated through the food supply (*12*–*19*).

The cefoperazone-containing medium routinely used in our laboratory for the isolation of *Campylobacter* occasionally yields other bacteria with hyperproduction of chromosomal β-lactamases or their plasmidic derivatives, as well as strains carrying extended-spectrum β-lactamases (unpub. data). By using this media, we have isolated several resistant enterobacteriaceae strains from patients with sporadic cases of gastroenteritis (unpub. data). During an investigation of a summer camp-associated salmonellosis outbreak, we observed that stool cultures from nine campers unexpectedly yielded, on cefoperazone-containing medium, colonies resembling enterobacteriaceae, with a uniform mucoid appearance. This result suggested the possibility that the same, probably ESC-resistant, enterobacterial strain was present in all these persons, findings consistent with possible foodborne spread. Consequently, all samples were reevaluated on media containing cefotaxime (see Methods) to increase sensitivity for detection of ESC-resistant organisms. To gain more knowledge of foodborne spread as a potential mechanism of dissemination of resistance genes, we undertook an extensive molecular epidemiologic analysis of these isolates.

## Methods

### Description of the Outbreak and Stool Sampling

Two hundred twenty-five elementary and secondary school students and 11 teachers were spending a week (June 11–15, 2001) at a summer camp in Palafrugell, Girona (Spain), when an outbreak of gastroenteritis began on June 14. An epidemiologic investigation involving 200 campers and staff failed to identify the source of the outbreak. Clinical and epidemiologic studies were initiated at the onset of the outbreak, but our participation as reference laboratory began later. Consequently, a limited number of stools from symptomatic patients and related persons were available to us for analysis.

From June 16 to 19, stool samples from 22 ill campers were collected for analysis. On July 6 (18–19 days later), additional stool samples were collected from four ill campers and from 10 asymptomatic household members of the four ill campers. Stool samples were also collected from three asymptomatic food handlers, on June 19, 22, and 28.

### Microbiologic Studies

#### Human Samples

Stool samples were processed according to conventional protocols for isolation of enteropathogenic bacteria, in addition to special selection for resistant organisms (as described below). Isolates were identified by conventional methods (*20*). Stools (fresh and after modified Ziehl-Neelsen staining) were microscopically examined for protozoa (*21*). Latex agglutination was used to detect rotavirus immunochrotography to dectect adenovirus 40/41, and reverse transcriptase–polymerase chain reaction (RT-PCR) (*22*) to detect calcivirus.

### Selection of ESCREC

Stool suspended in saline was added to trypticase soy broth containing 2 mg/L cefotaxime (TSB-CTX), which after 18 h of incubation at 35°C, was added to similarly supplemented MacConkey broth (MacC-broth-CTX). After an 18-h incubation, a loopful of this broth was spread on similarly supplemented MacConkey agar. A colony of each distinct morphotype was analyzed further.

### Selection of Resistant *S. enterica* Strains

Stool samples were processed as for isolation of ESCREC, but after the initial growth the MacC-broth-CTX was added to selenite broth. After 8 h of incubation at 35°C, this broth was spread on cefotaxime-supplemented xylose-lysine-deoxycholate agar. Colonies suspected of representing *Salmonella* were analyzed further.

### Food Studies

Eight unprepared food items from the camp kitchen were analyzed for *Salmonella* and ESCREC as described for stool samples, except that the initial TSB-CTX was replaced by peptone yeast extract broth.

### Antibiotic Susceptibility Testing

Disk diffusion susceptibility testing to 28 antibiotics (Table) was performed according to National Committee for Clinical Laboratory Standards (NCCLS) guidelines (*23*). The activity of cefotaxime and ceftazidime, combined with clavulanate, was determined by E test (AB Biodisk, Solna, Sweden). MICs to β-lactam antibiotics were determined by broth microdilution (Sensititre, Trek Diagnostic Systems LTD, West Sussex, England), according to NCCLS guidelines (*24*,*25*).

**Table:** Characteristics of 15 extended-spectrum cephalosporin-resistant fecal *Escherichia coli* isolates derived from an outbreak of salmonellosis

Patient no. (age in y)	School^b^	Isolate	Isolation date	Biotype	Serotype	PFGE pattern (clone)	Plasmid profile	Southern blot pattern		β-lactamase	Associated resistance^d^
								*Pst*I	*Sma*I / *Hinc*II^c^		
P5^a^ (12)	EP	1	06-18-01	5671	NT	A	I a^e^	1	a	CTX-M-9	Tp, Sxt
P6^a^ (11)	EP	1	06-18-01	5671	NT	A	I b^e^	2	b	CTX-M-9	Tp, Sxt
P9^a^ (12)	SB	1	06-19-01	5671	NT	A	I c^e^	2	b	CTX-M-9	Tp, Sxt
P10^a^ (12)	SB	1	06-19-01	5671	NT	A	I a^e^	3	c	CTX-M-9	Tp, Sxt
		2	06-19-01	7775	O:86	B	II a^e^	4	d	CMY-2	Tp, Sxt, Cm, Fur
P12^a^ (11)	SB	1	06-19-01	5671	NT	A	I b^e^	3	b	CTX-M-9	Tp, Sxt
		2	07-06-01	5671	NT	A	I b^e^	3	b	CTX-M-9	Tp, Sxt
P14^a^ (12)	SB	1	06-19-01	1571	O:20	C	III	3	e	CTX-M-9 + TEM-1	Tp, Sxt, Km, Neo
P15 (11)	SB	1	06-19-01	0371	O:145	D	IV	3	f	CTX-M-9 + TEM-1	Tp, Sxt, Cm
P18^a^ (11)	SB	1	06-19-01	5671	NT	A	I b^e^	3	b	CTX-M-9	Tp, Sxt
		2	06-19-01	7775	O:86	B	II b^e^	4	d	CMY-2	Cm, Fur
P19^a^ (12)	T	1	06-19-01	5671	NT	A	I b^e^	3	b	CTX-M-9	Tp, Sxt
F2		1	06-22-01	1571	O:20	C	V	4	nd^f^	CTX-M-9 + TEM-1	Tp, Sxt, Km, Neo
F3		1	06-22-01	4571	O:55	E	VI	4	d	CMY-2 + TEM-1	Cm, Gm, Km, Neo, Tob
		2	06-28-01	4571	O:55	E	VI	4	d	CMY-2 + TEM-1	Cm, Gm, Km, Neo, Tob

^a^*Salmonella enterica* also was isolated. ^b^SB, Sant Boi; T, Tarragona; EP, El Prat. ^c^The *Sma*I RFLP pattern was determined for those strains carrying the CTX-M-9 enzyme and the *Hinc*II RFLP pattern was determined for those carrying the CMY-2 enzyme. ^d^All ESCREC isolates were resistant to all penicillins and cephalosporins, including ESCs, as well as to nalidixic acid, tetracycline, sulfonamides and streptomycin. Other antibiotics tested included ampicillin, amoxicillin-clavulanate, piperacillin, cefazolin, cefuroxime, cefoxitin, cefotaxime, ceftazidime, cefepime, aztreonam, imipenem, chloramphenicol (Cm), gentamicin (Gm), kanamycin (Km), tobramycin (Tob), amikacin, neomycin (Neo), sulfamethoxazole-trimethoprim (Sxt), trimethoprim (Tp), ciprofloxacin, fosfomycin, nitrofurantoin, rifampin and furazolidone (Fur). ^e^The only difference between the Ia, Ib, and Ic, or the IIa and IIb plasmid profiles is one band higher than 100 kb within the particular plasmid pattern. ^f^nd, not determined.

### Transfer of Resistance Determinants

Filter matings were performed with ESCREC donors, using as recipients either *E. coli* HB101 (Nal Kan) for which transconjugants were selected, according to described methods (*26*), or the *S. enterica* isolate from patient P12, for which transconjugants were selected by adding the filter containing mixed growth of *E. coli* and *Salmonella* to MacC-broth-CTX. This broth, which was subsequently processed as described above for selection of resistant *S. enterica*.

### Extraction of β-Lactamases and Isoelectric Focusing (IEF)

Crude extracts of β-lactamases were obtained by ultrasonication. Analytical IEF was performed as previously described (*27*).

### Characterization of β-Lactamase Genes

The *bla*__TEM___,_ *bla*_CTX-M-9_ and *bla*_CMY-2_ genes were amplified as previously described (*28*–*33*). The DNA sequence was directly determined from PCR products for both DNA strands (*34*).

### Biotyping and Serotyping

The biotype, as determined for 12 metabolic reactions, was expressed as a 4-digit code (*35*). The serotype and phage type of *Salmonella* isolates were determined in the Servicio de Enterobacterias del Centro Nacional de Microbiología, Instituto Carlos III, Majadahonda, Spain (M.A. Usera and A. Echeita). The serogroup of the ESCREC was determined at the Laboratorio de Referencia, Lugo, Universidad de Santiago de Compostela (J. Blanco).

### Pulsed-Field Gel Electrophoresis (PFGE)

Genomic profiles were analyzed by PFGE with *Xba*I (Amersham Biosciences UK Limited, Buckinghamshire, England) (*36*,*37*). Isolates exhibiting indistinguishable PFGE profiles were considered to represent the same clone.

### Plasmid Profiles Analysis

Plasmid DNA was isolated by using a commercial kit (QIAGEN, Inc., Valencia, CA) and subjected to 0.8% agarose gel electrophoresis both without digestions and after cleavage with *Pst*I, *Sma*I, or *Hinc*II (Amersham Biosciences). For southern hybridization (*38*), both total and restricted plasmid DNA were transferred to nylon membranes and hybridized with PCR-generated probes for *bla_CTX-M-9_* (850 bp) and *bla_CMY-2_* (1,017 bp), as labeled and detected using the ECL^TM^ Direct Nucleic Acid Labeling and Detection System (Amersham Biosciences).

### Statistical Methods

Comparisons of proportions were tested by using the Fisher exact test (two-tailed).

## Results

### Epidemiologic Survey

The 225 student campers, 10–16 years of age, and 11 teachers were from three schools in three cities, Tarragona (T), El Prat (EP), and Sant Boi (SB). Of the 200 campers and staff interviewed, 109 (54.5%), including 3 teachers, had symptoms of gastroenteritis, with no significant differences between the three schools (57%, 49%, and 62% respectively). The most frequent symptoms were abdominal pain (80%), diarrhea (79%), and headache (64%). Two students were admitted to hospital, but after supportive therapy were discharged within 48 hours. No person received antibiotic therapy.

### Microbiologic Study

#### Campers

Of the 109 ill campers, 22 provided stool samples that were available for microbiologic study in our laboratory. Nineteen (86%) of the acute-phase stool samples yielded *S. enterica* serovar Enteritidis phage type 4, which was susceptible to all tested antimicrobial agents. No fecal pathogens were detected in the remaining three ill campers.

In addition, ESCREC were isolated from the initial stool sample for 9 (41%) of the 22 campers, including 8 (42%) of 19 with salmonellae and 1 with no detectable enteric pathogen (Table). All nine samples contained an ESCREC isolate resistant to penicillins and cephalosporins (including ESCs) but susceptible to cephamycins and carbapenems (CTX^R^-cefoxitin FOX^S^-ESCREC), consistent with production of an ESBL. Two of these samples also yielded a second ESCREC type that was resistant to penicillins and cephalosporins, including ESCs and cephamycins, but susceptible to carbapenems (CTX^R^-FOX^R^-ESCREC), consistent with hyperproduction of *E. coli* AmpC chromosomal β-lactamase or presence of a plasmidic cephamycinase. All ESCREC isolates exhibited multiple additional resistance markers (Table).

Follow-up stool samples (collected 18–19 days later) were available for four campers. Neither salmonellae nor ESCREC was recovered, except in one camper, from whom the initial ESCREC strain was isolated (Table).

### Food Handlers and Households Contacts

Although stool samples collected from the three (asymptomatic) food handlers were negative for enteropathogens, two of these persons were carriers of ESCREC. One had CTX^R^-FOX^S^-ESCREC at the second sampling, whereas the other had CTX^R^-FOX^R^-ESCREC at both the second and third samplings.

In contrast, neither enteropathogens nor ESCREC were detected in stool samples from 10 healthy household members of four ill campers who had both ESCREC and salmonellae in their acute-phase stool sample (for prevalence of ESCREC among household members vs. campers or food handlers, p=0.03 and p=0.04, respectively).

The eight cultured camp food items yielded neither *S. enterica* serovar Enteritidis nor ESCREC.

### *E. coli* β-Lactamases

In the 11 *E. coli* isolates phenotypically suspected of ESBL production, a β-lactamase with an isoelectric point of 8.0 was detected. PCR with *bla*_CTX-M-9_-specific primers and sequence analysis confirmed the presence of the CTX-M-9 β-lactamase (not shown).

Three of these isolates had an additional β-lactamase with pI 5.4, which in IEF reacted with penicillin but not with ceftriaxone. PCR with *bla*_TEM_-specific primers confirmed the presence of a TEM-1-like β-lactamase (Table).

All four isolates phenotypically suspected of AmpC β-lactamase production were PCR-positive with *ampC*-family primers. Sequence analysis confirmed the presence of the CMY-2 β-lactamase (not shown).

### Biotyping, Serotyping, and PFGE Analysis of Isolates

**Figure 1:**
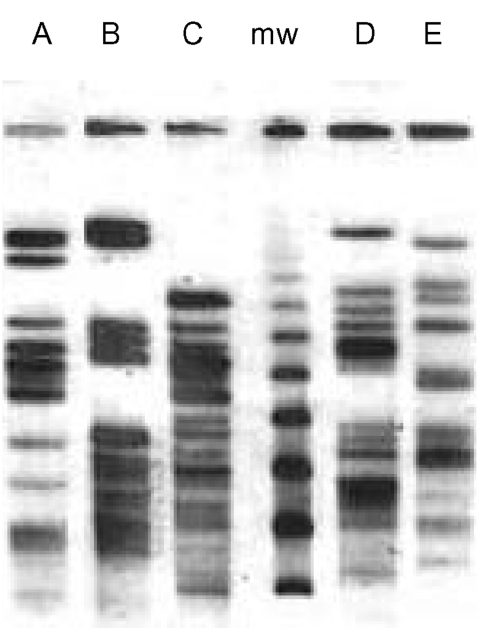
Restriction pattern (*Xba*I) by pulsed-field gel electrophoresis of the five extended-spectrum, cephalosporin-resistant *Escherichia coli* clones (A, B, C, D, E). mw: marker.

Collectively, the 15 ESCREC isolates represented five distinct biotypes (Table). Overall, five serogroups (O20, O55, O86, O145, and non-typable/NT) and five PFGE pulsotypes (A to E) were detected. Biotype, serogroup, and pulsotype corresponded precisely, confirming the presence of five discrete ESCREC clones (Table and Figure 1).

### Epidemiologic Distribution of Clones

Clone A (CTX-M-9), the most prevalent, was recovered from students from all three cities, EP, SB, and T (Table). Clone B (CMY-2) was recovered from two students from Sant Boi and clone E (also CMY-2) was recovered from a food handler. Clone C (CTX-M-9 + TEM-1) was recovered from both a Sant Boian student and a food handler. Clone D (CTX-M-9 + TEM-1) was recovered from a Sant Boian student.

### Plasmid Analysis

**Figure 2:**
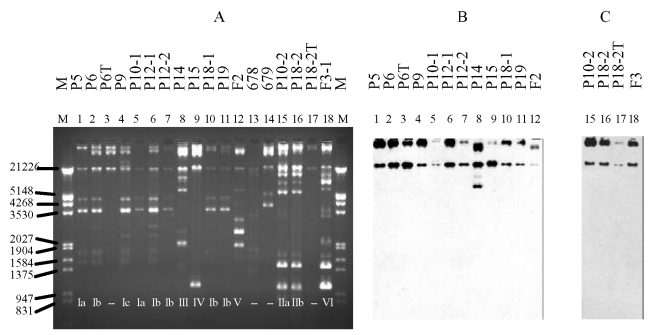
Plasmid profile (A) and hybridization with CTX-M-9 probe (B) and CMY-2 probe (C). The studied isolates, by lane, are: 1: P5, 2: P6, 3: P6T (transconjugant of P6), 4: P9, 5: P10-1, 6: P12-1, 7: P12-2, 8: P14, 9: P15, 10: P18-1, 11: P19, 12: F2, 15: P10-2, 16: P18-2, 17: P18-2T (transconjugant of P18-2), 18: F3-1, 13, and 14: plasmid control strains *E. coli* 678 CECT (= NCTC 50193 with the following plasmid sizes: 54.38, 7.30, 5.56, 5.14, 3.98, 3.08, 2.71, and 2.06 kb) and *E. coli* 679 CECT (= NCTC 50192 with the following plasmid sizes: 148.5, 63.8, 36.2 and 7 kb.). M is marker III (Roche Diagnostics GmbH, Mannheim, Germany). Below each lane in panel A is indicated the plasmid profile designation shown in the Table.

Plasmid profiles of the ESCREC isolates were largely concordant with clonal assignments (Figure 2A, Table). In all but one isolate, the probes for *bla_CTX-M-9_* and *bla_CMY-2_* hybridized to a single large (>150 kb) plasmid (Figure 2).

**Figure 3:**
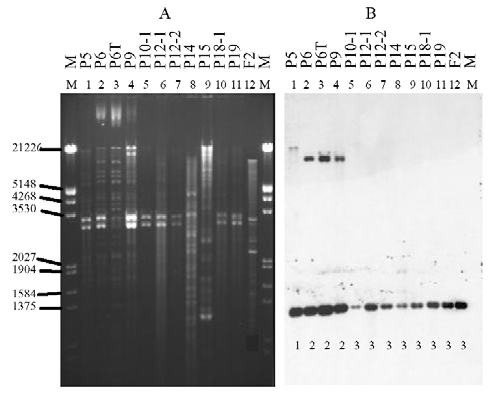
Plasmid restriction with *Pst*I (A) and hybridization of isolates carrying the CTX-M-9 enzyme with the CTX-M-9 probe (B). The isolates are as listed in Figure 1. Below each line of hybridization, the pattern shown in the Table is indicated.

**Figure 4:**
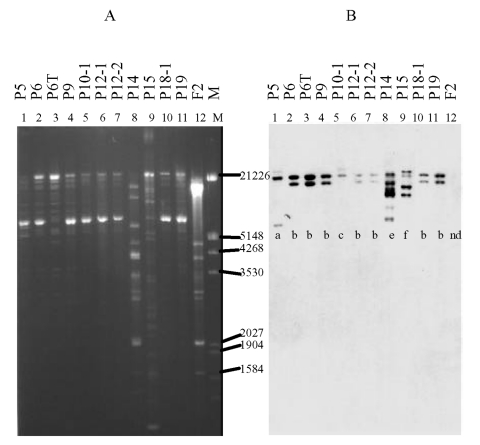
Plasmid restriction with *Sma*I (A) and hybridization of isolates carrying the CTX-M-9 enzyme with the CTX-M-9 probe (B). The isolates are as listed in Figure 1. Below each line of hybridization, the pattern shown in the Table is indicated.

By Southern blot analysis, the *bla_CTX-M-9_*-containing plasmids (clones A, C, and D) exhibited minor diversity within and greater diversity among clones (Figures 2–4, Table). In the uncut plasmid blot, the large, probe-positive plasmids of clones A and D were uniform in size and larger than those of clone C (Figure 2B). The corresponding *Pst*I Southern blot showed three different patterns, which were not clone-specific (Figure 3, Table). All of these isolates exhibited a band at ~1,289 bp (Figure 3B), whereas three isolates (all clone A) also exhibited a variably sized larger band (Figure 3B, Table). The corresponding *Sma*I blot showed five patterns among the CTX-M-9-positive isolates, including three closely related patterns among the clone A isolates and unique patterns each for the clone C and D isolates (Figure 4, Table).

In contrast, the *bla_CMY-2_*-containing isolates (clones B and E) were indistinguishable in both the uncut plasmid blot (Figure 2C) and the *Pst*I and *Hinc*II blots, which showed a homogeneous single-band pattern for all three isolates (not shown).

### Transfer of Resistance in vitro

One CTX-M-9-positive isolate transferred its resistance by in vitro conjugation to *E. coli* HB101 but not to a *S. enterica* isolate from a patient. In contrast, a CMY-2-positive isolate was successfully conjugated with this *S. enterica* isolate but not with *E. coli* HB101 (probably due to the donor strain’s production of a bacteriocin that inhibits HB101; data not shown).

## Discussion

Our microbiologic evaluation of an outbreak of *Salmonella* gastroenteritis at a summer camp uncovered the unsuspected dissemination among campers and camp staff of multiple clones of ESCREC containing diverse conjugally transferable β-lactamases. Several lines of evidence indicated that dissemination of ESCREC occurred within the summer camp. Sharing of ESCREC clones was observed among multiple hosts who had no contact with one another before camp, yet at camp lived together and shared a common food and water supply. Isolation of ESCREC was limited to camp attendees, to the exclusion of members of the campers’ households who did not attend camp. Finally, the high observed prevalence of fecal ESCREC among camp attendees (11/25, 44%) contrasts strikingly with the low prevalence of ESCREC detected by using similar methods in reference fecal samples from 707 outpatients without an infectious disease diagnosis who attended Sant Pau Hospital from February through May, 2001 (2%: p<0.001; unpub. data) and among *E. coli* isolates from our hospital clinical microbiology laboratory (e.g., in 2000, for CTX-M-9, 0.5%; for CMY-2, 0.2%) (*29*).

The mechanism for dissemination of ESCREC within the camp remains undefined. Direct person-to-person spread is possible but seems unlikely, since there was no evidence of transmission to household members after campers returned home was not evident, as would be expected if domestic contact could lead to transmission. However, hygienic conditions conceivably were worse at the camp, particularly during the outbreak of gastroenteritis.

The concurrent outbreak of salmonellosis, a classic foodborne pathogen, suggested that contaminated food (or possibly water) might have served as a vehicle for ESCREC within the camp. Indeed, ESCREC clones A and B were confined to hosts who also had salmonellae. Since only eight food items were cultured, the failure to recover ESCREC from camp foods provides little evidence to rule out foodborne transmission.

Although food handlers have been implicated in many foodborne outbreaks of intestinal disease (*39*), in this instance they appeared an unlikely source for either ESCREC or salmonellae. None of the three food handlers was colonized with salmonellae or with ESCREC clones A, B, or D, all of which were present in one or more campers, whereas one food handler had a unique ESCREC clone (clone E), one shared a distinct ESCREC clone (clone C) with a single camper, and one had no detectable ESCREC. Thus, if food were the vehicle, the contamination most likely occurred before the food’s arrival at the camp, i.e., during production, processing, or transport. Since CMY-2 is closely associated with food animals, the present CMY-2-positive ESCREC plausibly could be of food animal origin. In contrast, CTX-M-9 and other ESBLs have been described only in humans. Thus, their presence suggests a human source of contamination.

Plasmid analysis indicated that although distinctive CTX-M-9-encoding plasmids were present in clones A, C, and D, the constituent *bla_CTX-M-9_* genes clearly derived from a common source, as demonstrated by their internal sequence identity and conserved flanking *Pst*I sites, despite the diversity of flanking *Sma*I sites. Minor within-clone diversity was evident among the *bla_CTX-M-9_*-containing plasmids of clones A and C, consistent with recent microevolution. In contrast to the CTX-M-9-positive clones, clones B and E appeared to share the same *bla_CMY-2_*-containing plasmid, consistent with recent horizontal transfer. Since clone B was isolated from two different hosts, it presumably acquired the CMY-2 plasmid before dissemination. By contrast, since clone E was recovered from only one host, the timing of its acquisition of the plasmid could not be determined. The diffusion of indistinguishable plasmids between different clones and the presence of similar but distinct plasmids within the same clone indicate the rapid biological dynamics of plasmids (*9*,*10*).

Conjugal transfer in vitro of broad-spectrum β-lactamases was achieved from ESCREC isolates to both a laboratory strain of *E. coli* (CTX-M-9) and an outbreak *S. enterica* isolate (CMY-2). These findings, which are consistent with our previous work and that of others (*30*,*31*), suggest that intraspecies or intergeneric transfer of broad-spectrum β-lactamases could occur in nature, either in vivo (in humans or animals) or in an inanimate reservoir (e.g., sewage or manure) (*9*,*10*). Thus, ESCREC may pose a threat both because of their direct potential for causing drug-resistant infections and because they can serve as vector of resistance elements for transmission to other pathogens or opportunistic microorganisms.

Our findings provide novel evidence of the dissemination of ESCREC among otherwise healthy persons. Moreover, in two persons ESCREC strains were documented to persist for at least 6 days or 17 days, suggesting possible establishment of stable colonization. Although no drug-resistant *E. coli* infections were known to have resulted from this dissemination, the data nonetheless suggest the possibility of widespread future emergence within the community of *E. col*i that is resistant to ESCs and of the responsible resistance genes, which could have substantial adverse health consequences (*4*,*5*).

Limitations of this study include the modest sample size (particularly for household members), short longitudinal follow-up, unusual circumstances (concurrent salmonellosis outbreak, summer camp setting), and absence of data regarding prior antibiotic use and hospital contact. Future studies should seek ESCREC in a similar setting, and in the general community, during a period without a known infectious disease outbreak.

In summary, our microbiologic evaluation of an outbreak of *Salmonella* gastroenteritis at a summer camp showed the unsuspected dissemination among campers and staff of multiple clones of ESCREC that contained diverse, conjugally transferable β-lactamases. Dissemination of ESCREC within the summer camp, possibly through food or water, was suggested by several lines of evidence. Confirmation of community-based transmission of ESCREC in other contexts and locales would indicate a need for heightened vigilance and efforts to discover the reservoirs and vehicles for dissemination of ESCREC within the community.
